# Seminal plasma induces the expression of IL-1α in normal and neoplastic cervical cells via EP2/EGFR/PI3K/AKT pathway

**DOI:** 10.1186/1750-2187-9-8

**Published:** 2014-08-08

**Authors:** Anthonio O Adefuye, Kurt J Sales, Arieh A Katz

**Affiliations:** 1 MRC/UCT Receptor Biology Unit, Institute of Infectious Disease and Molecular Medicine and Division of Medical Biochemistry, University of Cape Town, Faculty of Health Sciences, Private bag X3 Observatory 7935, Cape Town 7925, South Africa

**Keywords:** Cervical cancer, Seminal plasma, Interleukin-1 alpha, EP2/EGFR/PI3kinase-Akt signaling pathways

## Abstract

**Background:**

Cervical cancer is a chronic inflammatory disease of multifactorial etiology usually presenting in sexually active women. Exposure of neoplastic cervical epithelial cells to seminal plasma (SP) has been shown to promote the growth of cancer cells in vitro and tumors in vivo by inducing the expression of inflammatory mediators including pro-inflammatory cytokines. IL-1α is a pleotropic pro-inflammatory cytokine induced in several human cancers and has been associated with virulent tumor phenotype and poorer prognosis. Here we investigated the expression of IL-1α in cervical cancer, the role of SP in the regulation of IL-1α in neoplastic cervical epithelial cells and the molecular mechanism underlying this regulation.

**Methods and results:**

Real-time quantitative RT-PCR confirmed the elevated expression of IL-1α mRNA in cervical squamous cell carcinoma and adenocarcinoma tissue explants, compared with normal cervix. Using immunohistochemistry, IL-1α was localized to the neoplastically transformed squamous, columnar and glandular epithelium in all cases of squamous cell carcinoma and adenocarcinomas explants studied. We found that SP induced the expression of IL-α in both normal and neoplastic cervical tissue explants. Employing HeLa (adenocarcinoma) cell line as a model system we identified PGE_2_ and EGF as possible ligands responsible for SP-mediated induction of IL-1α in these neoplastic cells. In addition, we showed that SP activates EP2/EGFR/PI3kinase-Akt signaling to induce IL-1α mRNA and protein expression. Furthermore, we demonstrate that in normal cervical tissue explants the induction of IL-1α by SP is via the activation of EP2/EGFR/PI3 kinase-Akt signaling.

**Conclusion:**

SP-mediated induction of IL-1α in normal and neoplastic cervical epithelial cells suggests that SP may promote cervical inflammation as well as progression of cervical cancer in sexually active women.

## Background

In sub-Saharan Africa, cervical cancer is the most common cancer among women accounting for 22.2% of all cancer cases and also the leading cause of cancer related deaths in this region [1,2]. Cervical cancer is a disease of multifactorial etiology usually presenting in sexually active women. Recent findings have shown that sexual transmission and persistent infection of the cervical epithelium with high risk HPV is the single most common risk factor for disease development, accounting for approximately 50% of cases [3]. Other risk factors include, sexually transmitted infections (STIs) [4], immunosuppression, and multiple sexual partners [5]. The hallmark of disease pathogenesis is characterized by chronic inflammatory response in the presence of underlining neoplasia [6,7].

Characteristically regarded as response to tissue injury or pathogenic insult, chronic inflammation is typified by alterations to vascular, epithelial, and immune cell function [4]. Over the last decade, numerous experimental studies using gene-disruption and gene over-expression systems in cell lines, laboratory animals, and tissue explants have provided evidence to support the role of inflammation and inflammatory pathways in the pathogenesis and progression of various human cancers including cervical cancer [8-12]. The inflammatory milieu of most cancer microenvironment has been shown to consist of tumor cells, surrounding stromal, immune and inflammatory cells which all interact intimately to produce cytokines/chemokines, growth factors, and adhesion molecules in a bid to promote tumorigenesis and metastasis [13]. Of special relevance within this milieu are pro-inflammatory cytokines which are important mediators of chronic inflammatory responses, and have cardinal effects on malignant processes.

Interleukin 1α (IL-1α) is a pleotropic pro-inflammatory cytokine that belongs to the IL-1 family (IL-1α, IL-1β, and IL-1Ra) gene located on the long arm of chromosome 2 [14]. IL-1α possesses a wide range of inflammatory, immunologic and tumorigenic properties [15-17]. IL-1α is secreted by a variety of cells including monocytes, tissue macrophages, neutrophils, fibroblasts, smooth muscle cells, dendritic cells, and cervical epithelium [15,18,19]. Accumulative evidence suggests that IL-1α plays a crucial role in tumorigenesis. Within the cancer microenvironment, IL-1α has been shown to induce the expression of metastatic genes such as the matrix metalloproteinases (MMPs) and stimulate the production of angiogenic proteins and growth factors such as IL-8, IL-6, vascular endothelial growth factor (VEGF), tumor necrosis factor-α (TNF-α), and transforming growth factor-β (TGFβ) [16,20].

Human Seminal plasma (SP) is a complex organic fluid comprising of secretions of the cowper’s, littre, prostate, and the seminal vesicles [21]. Once deposited within the female reproductive tract (vagina and cervix) during unprotected coitus, SP has been shown to induce the expression of several pro-inflammatory cytokines including IL-1α [22-24]. In sexually active women, the molecular pathways and degree at which SP normally activates the expression of these pro-inflammatory components in any compartment of the female reproductive tract is poorly understood. SP has been shown to possess an abundance of pro-inflammatory prostaglandins (PG) [25] and we and others have shown that cervical cancer has up-regulated expression of PG receptors [9] which can be activated by both endogenous and SP-PG. In the present study, we investigated the role of SP in the regulation of IL-1α expression in normal and neoplastic cervical epithelial cells and the molecular mechanism underlying this regulation.

## Results

### IL-1α is up-regulated in cervical cancer

**Figure 1:**
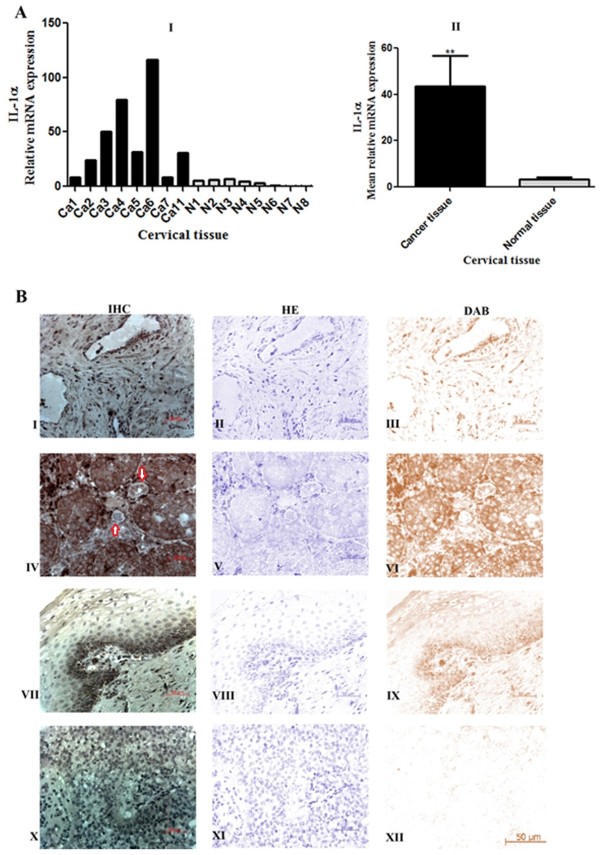
**Expression of IL-1α mRNA and protein in cervical squamous cell carcinoma, adenocarcinoma and normal cervix. A**, Relative mRNA expression of IL-1α in (I) cervical squamous cell carcinoma (Ca.1-Ca.7, adenocarcinoma (Ca.11), and normal cervix (N1-N8). (II) Mean relative IL-1α mRNA expression in neoplastic and normal cervical tissue explant as determined by qPCR and shown in (I). **B**, IL-1α expression by immunohistochemistry (IHC) staining. I, II, III shows IHC, hematoxylin (HE), and diaminobenzidine (DAB) stain of epithelial cells of squamous cell carcinoma, respectively. IV-VI shows IHC, HE, and DAB stain of columnar and glandular epithelium of adenocarcinoma, respectively. VII-IX shows IHC, HE, and DAB stain of normal tissue, respectively. Minimal IL-1α signal was detected in normal cervical tissue (VII and IX). X-XII is control and shows IHC, HE, and DAB stain of normal tissue, respectively. IL-1α staining was abolished in sections incubated with the biotinylated secondary antibody alone (negative control) (X and XII). Scale bar, 50 μm. Data represented as mean ± SEM. **, indicates statistical significance at P < 0.01.

Prior studies have shown that a major agonist protein of the interleukin 1 family (i.e. IL-1α) is present in abundance in tumor microenvironment where it plays a major role in tumourigenesis [14]. In this study, we initially investigated the expression of IL-1α in normal and neoplastic cervical tissue explants using real-time quantitative RT-PCR (qPCR) (Figure 1A I). Expression of IL-1α transcript was significantly elevated in all cancer tissue samples investigated compared with normal cervical tissue sample. IL-1α mean relative expression as assessed by qPCR was 43.65 ± 13.21 fold in neoplastic cervical tissue, while in normal cervical tissue it was 3.488 ± 0.911 (Figure 1A II). The site of IL-1α synthesis in the neoplastic tissue was then investigated by immunohistochemistry. IL-1α was localized to the neoplastically transformed squamous epithelium in squamous cell carcinoma (Figure 1B I) and to neoplastically transformed columnar epithelium lining the endocervical and the glandular epithelium of the endocervical glands in adenocarcinomas (Figure 1B IV). In addition, IL-1α immunostaining was observed in endothelial cells lining the vasculature in all adenocarcinoma tissue sections investigated (red arrows in Figure 1B IV). In contrast to the high levels of immunoreactive IL-1α observed in cervical cancer tissue, little or no staining for IL-1α was observed in the normal cervical tissues (Figure 1B VII). Incubation with biotinylated IgG in place of primary antibody as control did not show any immunostaining in carcinoma tissue sections (Figure 1B X).

### Seminal plasma induces expression of IL-1α in HeLa neoplastic cervical epithelial cells

**Figure 2:**
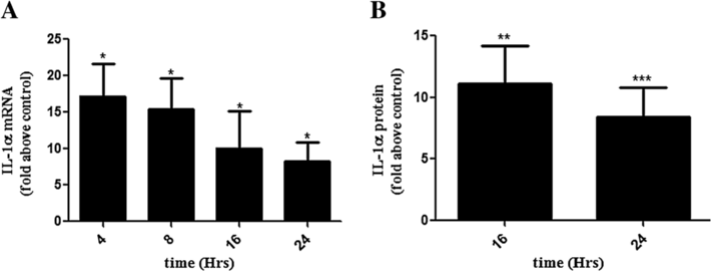
**SP induces IL-1α mRNA and protein expression in HeLa cells.** Relative expression of IL-1α mRNA **(A)** and IL-1α protein **(B)** in HeLa cells treated with SP (1:50 dilution) relative to PBS (control) for 4, 8, 16, or 24 hrs as determined by qPCR and ELISA. Data are presented as mean ± SEM from 5 **(A)** and 6 **(B)** independent experiments (n = 5 and 6). Paired T-tests were conducted on the untransformed means of the replicates between SP and control. *, **, and *** indicates significance at P < 0.05, P < 0.01 and P < 0.001, respectively.

SP has been shown to induce the expression of various inflammatory cytokines including IL-1α in normal cervical epithelial cells [24]. We hypothesized that SP can also induce IL-1α expression in neoplastic cervical epithelial cells, which could potentially enhance cervical inflammation and tumorigenesis. We used a well-established HeLa (adenocarcinoma) cell line as a model system to investigate the regulation of IL-1α expression by SP in neoplastic cervical epithelium. HeLa S3 cells were treated with vehicle or SP (1:50 dilution) for 4, 8, 16, and 24 hours and the expression of IL-1α mRNA assessed using qPCR (Figure 2A). SP significantly induced the expression of IL-1α mRNA in HeLa S3 cells at all-time point investigated. Peak induction of IL-1α was observed after 4 hours of SP exposure and was 17.02 ± 4.43 fold increase, while other inductions were 15.40 ± 4.26, 10.07 ± 5.03, and 8.22 ± 2.68 fold increase for 8, 16 and 24 hours, respectively.In addition, we investigated IL-α protein expression in response to SP treatment. Protein lysates extracted from HeLa S3 cells treated with vehicle or SP (1:50) for 16 and 24 hours, respectively were subjected to ELISA. We found that SP treatment of HeLa S3 cells significantly induced the expression of IL-1α protein compared to the control (Figure 2B; 10.91 ± 2.61 and 8.45 ± 2.36 fold increase for 16 and 24 hours, respectively). The ELISA did not detect IL-1α expression in lysate of cells treated with SP at earlier time points (4 and 8 hours).

### EP2 receptor antagonist and inhibitors of EGFR and PI3 kinase inhibit SP-mediated induction of IL-1α in HeLa neoplastic cervical epithelial cells

**Figure 3:**
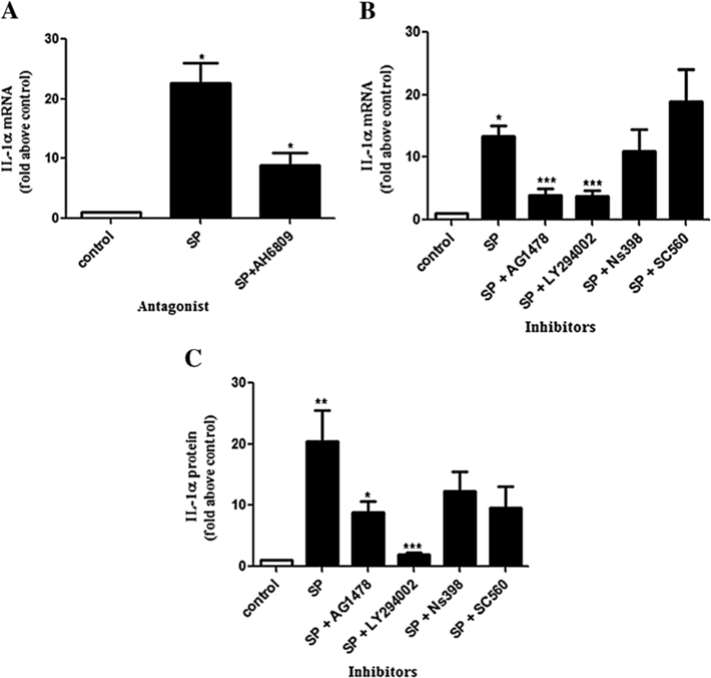
**IL-1α mRNA and protein expression in HeLa cells is regulated by seminal plasma via the EP2 receptor, EGFR and PI3K pathways.** IL-1α mRNA **(A** and **B)** and protein **(C)** as determined by qPCR and ELISA analysis, respectively. HeLa cells were treated for 4 **(A** and **B)** and 16 hours **(C)** with seminal plasma (1:50) or vehicle in the absence/presence of EP2 receptor antagonist [AH-6809; 20 μM] and chemical inhibitors to EGFR kinase [AG-1478; 100 nM], PI3 kinase [LY-294002; 25 μM], PTGS1 [SC-560; 15 μM] or PTGS2 [NS-398; 8 μM]. Data are represented as mean ± SEM from 5 independent experiments. Paired T-tests were conducted on the untransformed means of the replicates between SP and control and unpaired T-tests performed on SP versus SP and inhibitor after conversion to fold increases. *, **, and *** indicates significance at P < 0.05, P < 0.01, and P < 0.001, respectively.

We next investigated the signal transduction pathways mediating SP induction of IL-1α mRNA expression, using PGE receptor 2 (EP2) antagonist and a panel of small molecule chemical inhibitors of signaling proteins. HeLa S3 cells were treated with vehicle or SP (1:50) alone or with EP2 receptor antagonist (AH-6809) or chemical inhibitors of EGFR (AG-1478), PI3 kinase (LY-294002), PTGS1 (SC-560), and PTGS2 (NS-398) for either 4 hours or 16 hours. The expression of IL-1α in HeLa S3 cells following SP treatment in the presence of receptor antagonist or chemical inhibitors was determined by qPCR after 4 hours (Figure 3A and B) and ELISA after 16 hours (Figure 3C). Figure 3A shows that EP2 receptor antagonist significantly reduced SP-mediated induction of IL-1α mRNA in HeLa S3 cells (P < 0.05). SP-mediated induction of IL-1α was also found to be markedly reduced in the presence of chemical inhibitors of EGFR (AG-1478) and PI3 kinase (LY-294002), respectively (Figure 3B; P < 0.001). Co-incubation of SP with selective PTGS1 (SC-560) and PTGS2 (NS-398) inhibitors had no inhibitory effect on SP-induced IL-1α mRNA expression (Figure 3B; P = 0.26 and 0.16, respectively). This indicates that SP-mediated induction of IL-1α mRNA expression is via EP2 and EGF receptors and not via the endogenous PTGS-PG pathway. In addition, co-treatment with EGTA [calcium chelator; 1.5 mM] and PD-98059 [ERK inhibitor; 50 μM] did not inhibit SP-mediated induction of IL-1α (data not shown).Similarly, data obtained from measuring IL-1α protein with ELISA (Figure 3C) revealed that AG-1478 and LY-294002 reduced the expression of SP-induced IL-1α protein in HeLa S3 cells from 20.42 ± 5.16 fold increase to 8.80 ± 1.89 and 2.00 ± 0.32 fold increases, respectively (P < 0.05 and P < 0.001, respectively). However no significant decrease in IL-1α protein secretion was seen in the presence of NS-398 and SC-560 as compared to SP (1:50) (Figure 3C; P = 0.17 and P = 0.11, respectively). These results together demonstrate that SP-mediated induction of IL-1α transcript and protein expression is via EP2 and EGF receptors and not via the endogenous PTGS-PG pathway.

### PGE_2_ and EGF induces IL-1α expression in HeLa neoplastic cervical epithelial cells via their cognate receptors

**Figure 4:**
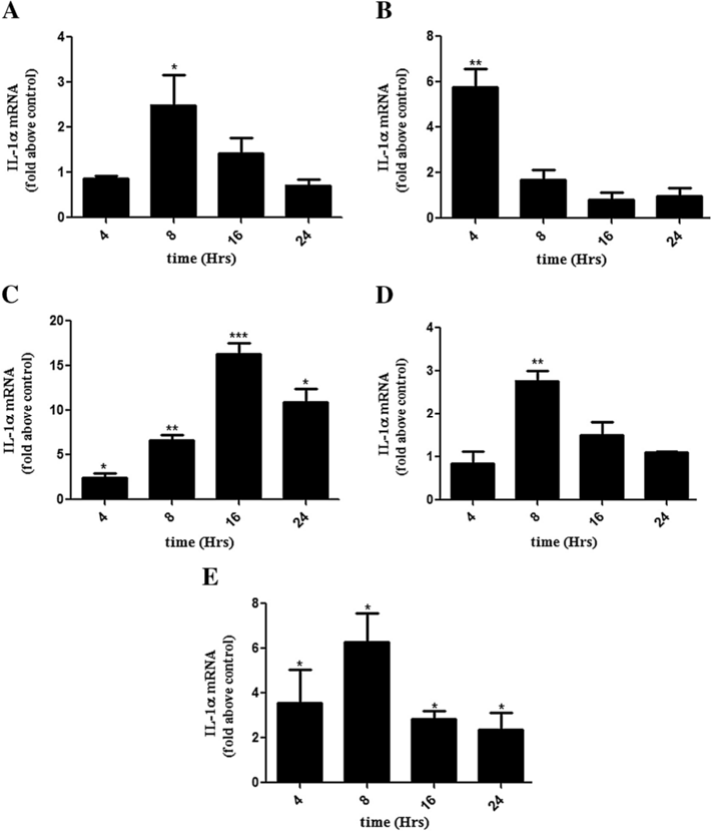
**Induction of IL-1α mRNA in HeLa cells by PGE2 and Butaprost with and without EGF.** Induction of IL-1α mRNA in HeLa cells by PGE_2_**(A)**, EGF **(B)**, PGE_2_ and EGF **(C)**, butaprost **(D)** and butaprost and EGF **(E)** as determined by qPCR. HeLa cells were treated for 4, 8, 16 and 24 hours with PGE_2_ [300 nM] or human recombinant EGF [10 ng/mL] or butaprost [5 μM] or butaprost together with EGF or PGE_2_ together with EGF or vehicle as control. Data are represented as mean ± SEM from 5 independent experiments. Paired T-tests were conducted on the untransformed means of the replicates between PGE_2_ or EGF or butaprost or butaprost and EGF or PGE_2_ and EGF treated cells and control. *, ** and *** indicates significance at P < 0.05, P < 0.01, and P < 0.001, respectively.

Human SP is a complex organic fluid comprising of a vast diversity of antigenically distinct molecules that include prostaglandins and growth factors [26,27]. We therefore hypothesized that PGE_2_ and EGF can induce IL-1α expression. To investigate the role of PGE_2_ and EGF on IL-1α expression, we treated HeLa cells with vehicle or PGE_2_ [300 nM] or human recombinant EGF [10 ng/mL] alone or together for 4, 8, 16, and 24 hours and determined IL-1α mRNA expression using qPCR. Treatment of HeLa cells with PGE_2_ (Figure 4A) and EGF (Figure 4B) resulted in a 2.49 ± 0.66 and 5.76 ± 0.80 maximum fold increase after 8 and 4 hours, respectively. Treatment of HeLa cells with both PGE_2_ and EGF together (Figure 4C) resulted in 2.39 ± 0.52, 6.60 ± 0.63, 16.31 ± 1.23 and 10.88 ± 1.52 fold increase after 4, 8, 16, and 24 hours, respectively. With peak IL-1α mRNA induction observed after 16 hours treatment (16.31 ± 1.23 fold increase). Furthermore, the inductions of IL-1α mRNA at 8, 16, and 24 hours by both ligands together was greater than by each ligand on its own and suggesting that PGE_2_ and EGF act synergistically in inducing the increase of IL-1α production by HeLa cells.The marked inhibition of SP-mediated induction of IL-1α by the EP2 receptor antagonist AH-6809 and EGFR kinase inhibitor AG-1478 indicated a role for EP2 receptor in combination with EGFR in this induction of IL-1α. We next investigated whether IL-1α regulation was mediated by the EP2 and EGF receptors. In order to confirm that activation of the EP2 and EGF receptors can regulate IL-1α expression, we treated HeLa cells with vehicle or butaprost which is a specific EP2 receptor agonist or human recombinant EGF or both agonist together for 4, 8, 16 and 24 hours. Activation of EP2 (Figure 4D) receptor significantly induced IL-1α expression after 8 hours treatment (2.77 ± 0.23) as shown by qPCR analysis. Co-treatment of HeLa cells with EP2 and EGF receptor agonists together (Figure 4E) significantly induced the expression of IL-1α mRNA at all-time points investigated (Figure 4E; 3.56 ± 1.48, 6.29 ± 1.28, 2.84 ± 0.34, and 2.36 ± 0.75 fold increases). Moreover, the peak IL-1α induction observed after 8 hours of co-treatment was greater than either butaprost or EGF treatment alone suggesting that co-activation of EP2 and EGFR act synergistically in inducing the increase of IL-1α production by HeLa cells (Figure 4E).

### EP2 receptor antagonist, EGFR and PI3 kinase inhibitors inhibit PGE_2_ and EGF mediated induction of IL-1α in HeLa neoplastic cervical epithelial cells

**Figure 5:**
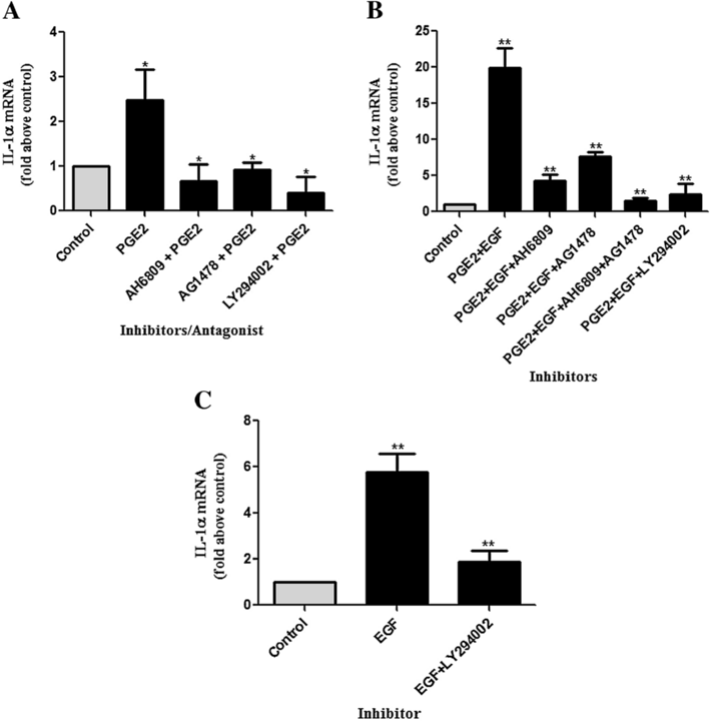
**EP2 antagonist and EGFR and PI3 kinase inhibitors, inhibit PGE2 or PGE2 and EGF or EGF mediated induction of IL-1α in HeLa cells.** EP2 antagonist, EGFR and PI3 kinase inhibitors inhibit PGE_2_**(A)**, PGE_2_ and EGF **(B)**, and EGF **(C)** mediated induction of IL-1α in HeLa cells as determined by qPCR. HeLa cells were treated for 4, 8 and 16 hours with EGF [10 ng/mL], or PGE_2_ [300 nM] or both or vehicle in the absence/presence of antagonist/inhibitors AH6809 [20 μM], AG1478 [100 nM], AH6809 together with AG1478, and LY294002 [25 μM]. Data are represented as mean ± SEM from 5 independent experiments. Paired T-tests were conducted on the untransformed means of the replicates between ligand/ligands treated cells and control and unpaired T-tests performed on ligand/ligands versus ligand/ligands and inhibitor after conversion to fold increases. * and ** indicates significance at P < 0.05 and P < 0.01, respectively.

Having demonstrated that PGE_2_ and EGF induced the expression of IL-1α in HeLa S3 cells, we next investigated the transduction pathways by which PGE_2_ and EGF induce IL-1α expression in HeLa cells. SP was shown to induce IL-1α expression in HeLa S3 cell via the EP2/EGFR/PI3 kinase pathways (Figure 3A, B and C), we thus investigated whether PGE_2_ and EGF which are abundant in SP induces the expression of IL-1α in HeLa cells via similar pathways. HeLa S3 cells were treated with vehicle or PGE_2_ alone or in the presence of AH-6809 and chemical inhibitors, AG-1478 or LY-294002 for 8 hours and IL-1α mRNA expression was assessed using qPCR (Figure 5A). PGE_2_-mediated induction of IL-1α (2.49 ± 0.66 fold increase) was significantly reduced in the presence of EP2 receptor antagonist (0.65 ± 0.38) and chemical inhibitors of EGFR and PI3 kinase (0.91 ± 0.15, and 0.41 ± 0.35 fold increases, respectively; P < 0.05 in all cases). Subsequently, HeLa S3 cells were treated with vehicle, or PGE_2_ and EGF alone or in the presence of AH-6809 or AG-1478 separately or together or with LY-294002 for 16 hours (Figure 5B). Similarly, induction of IL-1α after treatment with PGE_2_ and EGF together was markedly reduced in the presence of EP2 receptor antagonist or EGFR kinase inhibitor, and almost abolished in the presence of EP2 receptor antagonist together with EGFR inhibitor or in the presence of PI3 kinase inhibitor (P < 0.01 in all cases). In addition, HeLa S3 cells were treated with vehicle or EGF alone or in the presence of LY-294002 for 4 hours (Figure 5C). The EGF-mediated IL-1α induction was reduced from 5.76 ± 0.80 fold increase to 1.88 ± 0.46 fold increase in the presence of PI3 kinase inhibitor (P < 0.01). These findings confirm that EGF regulates IL-1α expression via the activation of PI3 kinase pathways, while PGE_2_ regulates IL-1α expression via the activation of EP2/EGFR/PI3 kinase pathways.

### Seminal plasma phosphorylates AKT via EP2/EGFR/PI3 kinase signaling to induce IL-1α expression in HeLa neoplastic cervical epithelial cells

**Figure 6:**
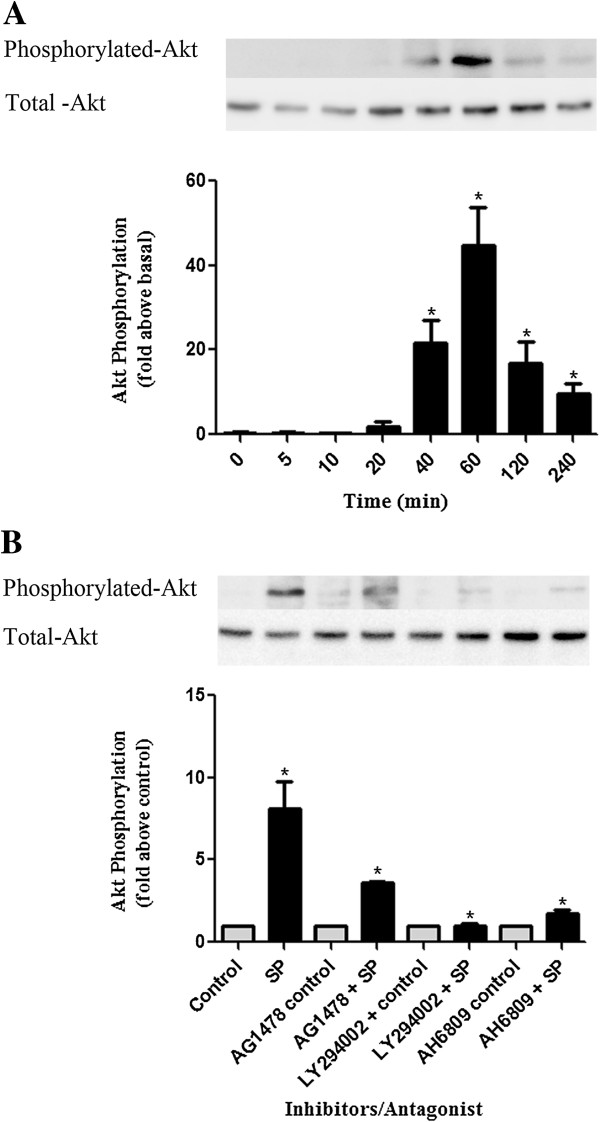
**SP induces Akt phosphorylation in HeLa cells. (A)** Akt phosphorylation in HeLa S3 cells treated with SP (1:50) or control for 0, 5, 10, 20, 40, 60, 120, and 240 min. **(B)** Akt phosphorylation in HeLa cells treated for 60 min with SP (1:50) or control in the presence/absence of chemical inhibitors/antagonist EGFR kinase [AG-1478;100 nM], PI3 kinase [LY-294002; 25 μM] and EP2 antagonist [AH-6809; 20 μM]. Cell lysate were subjected to immunoblot analysis. Data are represented as mean ± SEM form 3 independent experiments. Paired T-tests were conducted on the untransformed means of the replicates between SP and control and unpaired T-tests performed on SP versus SP and inhibitor after conversion to fold increases. *indicates P < 0.05.

Protein kinase B (Akt) is known to be one of the major downstream targets of PI3 kinase and upon activation, Akt moves to the cytoplasm and nucleus where it phosphorylates numerous downstream targets involved in the regulation of various cellular functions [28]. Having shown that SP mediated the expression of IL-1α in HeLa cells via the activation of EP2/EGFR/PI3 kinase pathways, we investigated the role of SP in the phosphorylation of Akt and the position of EP2, EGFR, and PI3 kinase pathways in relation to Akt signaling using immunoblot analysis. HeLa S3 cells were treated with vehicle or SP (1:50 dilution) for 0, 5, 10, 20, 40, 60, 120 and 240 minutes and Akt phosphorylation was measured by immunoblot analysis. A significant Akt phosphorylation was observed after 40 minutes with a maximum phosphorylation at 60 minutes and remaining until 240 minutes post-stimulation with fold increases of 21.58 ± 5.32, 44.64 ± 9.14 and 9.581 ± 2.475, respectively for these time points (Figure 6A) (P < 0.05). We next treated HeLa S3 cells with vehicle or SP (1:50 dilution) or in the presence or absence of chemical inhibitors of EGFR kinase (AG 1478), PI3 kinase (LY294002), and EP2 receptor antagonist (AH6809) for 60 min and measured Akt phosphorylation. SP-mediated phosphorylation of Akt was significantly reduced from fold increase of 8.08 ± 1.688 to fold increases of 1.70 ± 0.23, 3.62 ± 0.08, and 1.00 ± 0.16 in the presence of AH-6809, AG-1478, and LY-294002, respectively (P < 0.05) (Figure 6B).

### SP induces the expression of IL-1α in the cervix via EP2/EGFR/PI3 kinase activation

**Figure 7:**
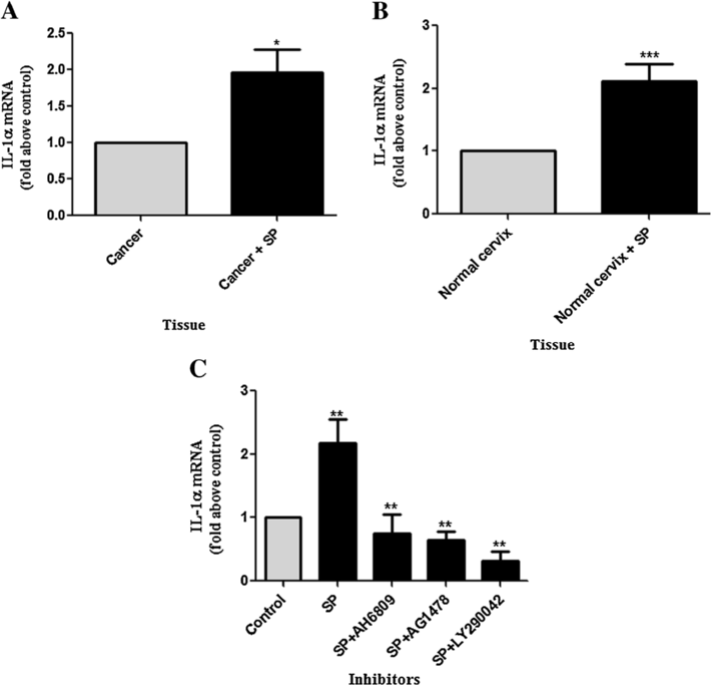
**SP induces IL-1α mRNA expression in neoplastic and normal cervical tissue explants.** SP induce IL-1α mRNA expression in neoplastic **(A)** and normal **(B)** cervical tissue explant. SP induces the expression of IL-1α in normal cervical tissue via the activation of EP2/EGFR/PI3 kinase inflammatory pathways as determined by qPCR **(C)**. Data represented as mean ± SEM of (n = 12 (A), n = 10 (B) and n = 7 (c), respectively). Paired T-tests were conducted on the untransformed means of the replicates between SP and control and unpaired T-tests performed on SP versus SP and inhibitor after conversion to fold increases. *, ** and *** represent significance at P < 0.05, P < 0.01 and P < 0.001, respectively.

Finally we investigated the potential role of SP in the regulation of IL-1α expression in the cervix and determined whether SP induces IL-1α expression in the cervix via the same pathways as seen in the HeLa model cell line. Normal and neoplastic (squamous) cervical tissue explant were treated with vehicle or SP (1:50) for 24 hours and IL-1α mRNA expression was determined using qPCR. SP significantly induced the expression of IL-1α in both neoplastic and normal cervical tissue with similar fold induction (1.959 ± 0.3226 and 2.124 ± 0.2673 fold increases) (Figure 7A and B). To investigate the signaling pathways by which SP induces IL-1α expression in the cervix, normal cervical tissue explants were incubated with vehicle or SP (1:50) in the presence or absence of AH-6809, AG-1478, and LY-290042. qPCR assessment of IL-1α expression showed a marked reduction in SP-mediated induction of IL-1α in normal cervical tissue explants in the presence of EP2 receptor antagonist or the chemical inhibitors of the EGFR (AG-1478) and PI3 kinase (LY-294002) pathways (P < 0.01 in all cases) (Figure 7B).

## Discussion

Inflammation is a characterized biological response of vascularized tissues to harmful stimuli, including chemical irritants or microbial pathogens [29]. Over the years, the role of inflammation as an etiological factor for cancer has been supported by findings that show that regular use of non-steroidal anti-inflammatory drugs (NSAIDs) is associated with reduced incidence of certain cancers [30]. Hence, inflammatory responses within tumor microenvironment are now recognized as a critical component for tumor progression and one of the major hall-marks of cancer [31,32]. Despite the numerous experimental and epidemiological evidence that supports the causal relationship between inflammation and cancer, the molecular mechanisms and pathways linking inflammation and cancer remain poorly understood [29]. The inflammatory milieu of most cancer microenvironments consist of various cells (tumor, surrounding stromal, immune and inflammatory) which all interact intimately to produce cytokines/chemokines, growth factors, and adhesion molecules in a bid to promote inflammation, tumorigenesis and metastasis [13]. Of special relevance within this milieu are pro-inflammatory cytokines which are important mediators of chronic inflammatory responses, and have cardinal effects on malignant processes [30] as a result of their direct involvement in carcinogenesis, malignant transformation, tumor growth, invasion, and metastasis [14].

Cervical cancer is a chronic inflammatory disease and one of the leading causes of cancer-related death worldwide with a higher incidence rate reported in underdeveloped countries [33]. It is well established that persistent infection with high-risk HPV is crucial to disease pathogenesis [34]. However, only a subset of women infected with high-risk HPV will proceed to develop invasive cervical cancer, thus suggesting that other co-factors must be present for the development of malignancy [7]. Studies have reported an association between the level of cervical inflammation and the development of high grade cervical neoplasia [35] or invasive cervical cancer [36]. It has been reported that cervical inflammation, but not the actual diagnosis of a specific sexually transmitted infection is associated with the development of squamous intraepithelial lesions within the cervix [37]. Direct links between increased pro-inflammatory cytokine levels in patients and increasing grade of cervical intraepithelial neoplasia and invasive cervical cancer have been established [38].

Using immunohistochemistry and qPCR, this present study investigated the expression of pro-inflammatory cytokine IL-1α in normal and neoplastic cervical tissue. Data presented confirmed the elevated expression of IL-1α in cervical cancer. This is in agreement with a similar study by Pao et al. (1995), where it was reported that neoplastic cervical tissue overtly expresses IL-1α [39]. In addition, these data suggests a similar pattern of IL-1α expression in cervical cancer as demonstrated in other malignancies [40-42]. IL-1α is a pleiotropic pro-inflammatory cytokine and a member of the IL-1 family. Within the human body, IL-1α mediates normal physiological functions ranging from induction of vascular permeability and fever during sepsis to increased secretion of additional cytokines in autoimmune diseases [30]. The production and level of IL-1α expression is elevated in numerous cancers including head and neck [43], breast [40], pancreatic [44], and gastric cancer [45] and has been associated with virulent tumor phenotype and poorer prognosis via the regulation of inflammatory genes and growth factors to enhance tumor growth and differentiation [46-48] and metastatic potential of cells [49]. Animal studies have further confirmed the role of IL-1α in tumor development and blood vessel growth [50]. Similarly, studies by Woodworth et al. [51] and Castrilli et al. [48] showed that IL-1α promotes in vitro growth and proliferation of both normal and human papillomavirus-immortalized and carcinoma-derived cervical epithelial cells. It is therefore probable that expression of IL-1α in cervical cancers can act via similar manner to confer virulent tumor phenotype and poorer prognosis in these patients.

IL-1α can be regulated by a host of inflammatory stimuli and recent studies have shown that seminal plasma can regulate IL-1α expression in the human cervix, post coitus [24,52]. Conventionally, human seminal plasma was regarded primarily as a transport and survival medium for the mammalian spermatozoa traversing the cervix and the uterus during and post coitus [26,53]. However, experimental studies using animal models shows that in addition to its role as a primary transport medium for the spermatozoa, seminal plasma also introduce to the female reproductive tract an array of antigenically distinct signaling molecules including prostaglandins, several cytokines and growth factors [26,53-55]. These molecules interact with cognate receptors on the epithelial lining of the female reproductive tract to initiate local cellular and molecular changes reminiscent of an inflammatory response [26]. These changes are required for maternal immune adaptation to pregnancy and for the generation of immune tolerance against fetal antigens [26,56]. However the molecular pathway by which seminal plasma mediates the expression of IL-1α and other cytokines is yet to be fully elucidated. Hence, using HeLa (adenocarcinoma) cells, normal and neoplastic cervical tissue explants this study investigated the role of SP in the regulation of IL-1α expression in the cervix and transduction pathways by which SP induces the expression of IL-1α in neoplastic and normal cervical epithelium. In addition this study investigated PGE_2_ and EGF as possible ligands mediating SP induction of IL-1α in neoplastic cervical cells.

In the present study we show that exposure of HeLa neoplastic cervical epithelial cells to SP in vitro increases the expression of IL-1α. In a similar manner, SP was found to increase the expression of IL-1α in both normal and neoplastic cervical tissue explants. This in agreement with studies by Sharkey et al. (2007 and 2012) where it was reported that SP after deposition into the female reproductive tract at coitus induces the expression of pro-inflammatory cytokines including IL-1α in the cervix [24,52] and initiates an inflammatory response. Hence it is likely that in sexually active women with underlying cervical pathology, recurrent inflammation consequent of SP-mediated IL-1α expression may enhance disease progression. Having shown that SP regulates IL-1α expression in the normal and neoplastic cervical tissue and epithelial cells, we next investigated possible signal transduction pathways by which SP mediates this role.

Employing HeLa cell line as a model, we discovered that SP induced the expression of IL-1α via the EP2 receptor, EGFR and PI3 kinase pathways since EP2 receptor antagonist (AH6809) and the inhibitors of EGFR kinase (AG1478) and PI3 kinase (LY294002) inhibited SP mediated induction of IL-1α in these neoplastic cells. In contrast, PTGS1 and PTGS2 were not shown to have a role in the induction of IL-1α by SP, since addition of their inhibitors (SC-560 and NS-398, respectively) did not reduce the induction of IL-1α. In addition, we found that SP-mediated induction of IL-1α in normal cervical tissue explant was also inhibited in the presence of the antagonist and these inhibitors. Similar in vitro studies by Battersby et al. (2006), Muller et al. (2006) and Sales et al. (2012) have shown that SP-mediated expression of pro-inflammatory and angiogenic genes in endometrial and cervical adenocarcinoma cells was significantly inhibited in the presence of AH6809 [57] and AG-1478 (EGFR kinase inhibitor) [58,59]. The inhibition of SP-mediated IL-1α by EP2 antagonist and EGFR kinase inhibitors suggested that the effects we observed were mediated by PGE_2_ and EGF present in the SP.

PGE_2_ has been established as the predominant PG found in SP [25]. In the present study, we show that PGE_2_-mediated activation of IL-1α occurs via activation of the EP2, EGFR and Akt pathways. The role of the EP2 receptor in mediating these effects was further confirmed using the selective EP2 agonist butaprost. This is consistent with similar study by Shao et al. (2007) where it was shown that PGE_2_, acting via EP2 receptor activate cAMP/PKA pathway to mediate the expression of IL-1α in colon cancer cells in an autocrine/paracrine mechanism [18]. EP2 and its signaling have been found to be up-regulated in cervical cancer [9] and its role in the induction of IL-1α in cervical cancer could explain the greater expression of IL-1α in cervical cancer tissue explant relative to normal cervical tissue. These data suggest that PGE_2_ in SP [26,60,61] can act via its E-series PGs receptor EP2 receptor to directly transactivate EGFR via an intracellular signaling mechanism, either by phosphorylation of cSRC or by the MMP-mediated release of heparin-bound EGF tethered to the cell membrane, [57,59] leading to IL-1α induction. In addition to PGE_2_, SP has been shown to be rich in growth factors including epidermal growth factor (EGF) [62-64]. Data presented herein showed that EGF can induce IL-1α in neoplastic cervical HeLa cells. This in agreement with similar in vitro study by Hamilton et al. (2003) where it was shown that EGF induces the expression of pro-inflammatory cytokine in lung cancer cells, and the expression of this cytokine was suppressed in the presence of EGFR inhibitor (AG-1478) [65]. It is therefore very feasible that the EGFR expressed on the membrane of these neoplastic cells can be directly activated by EGF in SP to induce IL-1α expression. The evidence that IL-1α induction by SP is due to PGE_2_ and EGF present in SP is in agreement with findings of Sharkey et al. (2012) who demonstrated that SP-mediated IL-1α induction in Ect1 cells occurred independently of TGF-β_1_, TGF-β_2_, and TGF-β_3_ which are abundant in SP [66].

Furthermore, our data show that concurrent treatment of HeLa S3 cells with PGE_2_ and EGF directed a sustained and elevated increase in IL-1α expression compared to either ligand alone or butaprost and EGF. Prior study by Sales et al. (2002) showed that PGE_2_ significantly induces the expression of EP4 receptor in HeLa cells [67], increased expression of EP4 receptor in the presence of EGF could explain the sustained and elevated increase in IL-1α expression mediated by PGE_2_ and EGF in these cells. Furthermore, transactivation of EGFR via PGE_2_-EP2 and PGE_2_-EP4 signaling could also augment EGF induction. In addition, PGE_2_ has been shown to induce the expression of amphiregulin [68], a ligand for EGFR and activates EGFR signaling. Taken together, it is likely that the effects of SP on IL-1α induction may be mediated by a combination of PGE_2_ and EGF working in synergy. Once released, IL-1α can act in an autocrine/paracrine manner within the site of production to regulate inflammation and tumorigenesis. Indeed, Shao and colleagues showed in their study that IL-1α stimulates the migration of colon cancer cells [18]. It is therefore plausible that in sexually active women with underlying pre-invasive or invasive cervical condition, repeated exposure of the elevated EP2 receptor expressed on the neoplastic cervical epithelial cells [9] to PGE_2_ present in seminal plasma could enhance tumorigenesis following ligand-receptor binding and activation of similar intracellular signaling pathway to induce IL-1α expression. Expressed IL-1α can then stimulate cervical cancer cell migration to adjacent structures within the pelvis and perineum, hence conferring poor prognosis.

Several studies have shown that PI3 kinase-Akt signaling is deregulated in many cancers including cervical cancer where amplification of the p110α catalytic subunit has been reported [69-72]. Interestingly in this present study, SP and its constituents (PGE_2_ and EGF) have been shown to mediate IL-1α expression in neoplastic cervical epithelial cells via the activation of PGE_2_-EP2-EGFR-PI3 kinase pathways. Once activated Akt phosphorylates proteins on serine and threonine residues resulting in the modulation of multiplicity of downstream substrates, including NF-κB involved in the regulation of cell proliferation and survival [73-75]. The SP-mediated Akt phosphorylation seen in this study may act via similar mechanism to induce IL-1α expression. The SP-mediated induction of a pleotropic pro-inflammatory cytokine IL-1α in normal and neoplastic cervical epithelial cells suggests that SP may promote cervical inflammation as well as progression of cervical cancer in sexually active women [58]. Furthermore, this present study is the first to demonstrate that SP regulates pro-inflammatory cytokine IL-1α expression in normal and neoplastic cervical cells via the induction of the EP2-EGFR-PI3 kinase-Akt pathways.

## Conclusion

**Figure 8:**
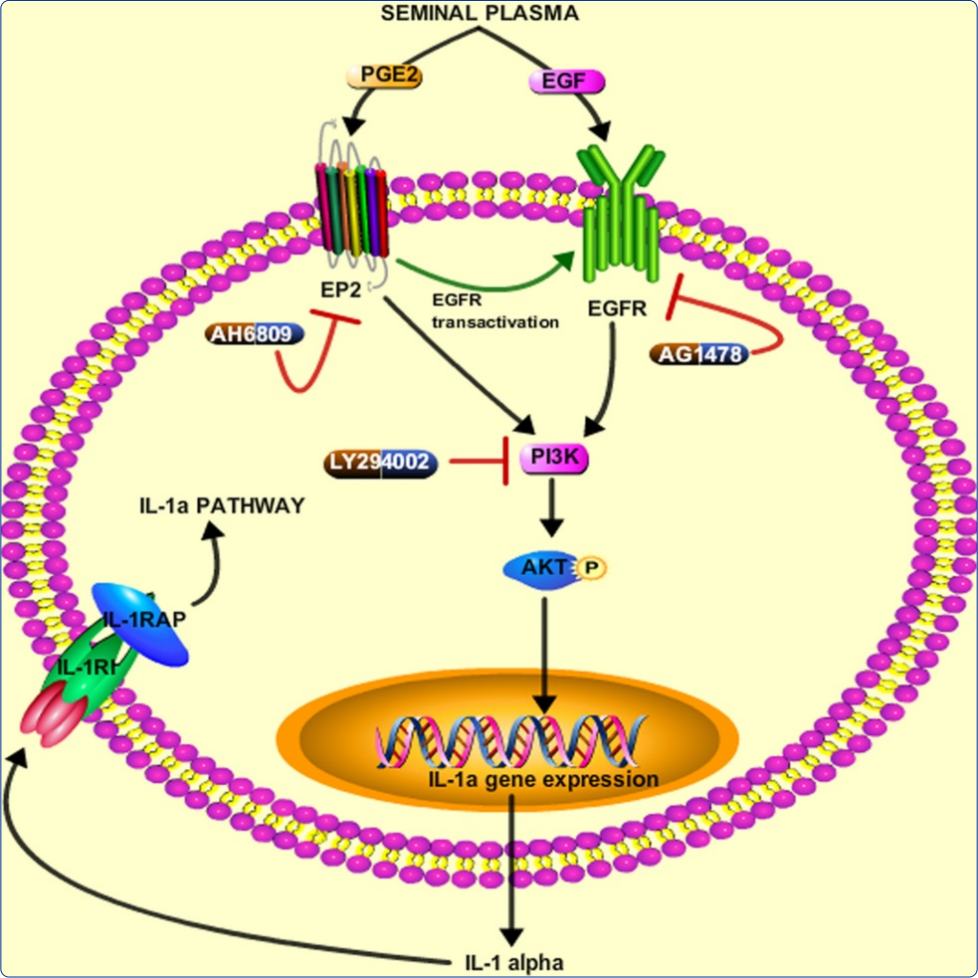
**Schematic summary highlighting the role of SP and its constituents (PGE**_**2 **_**and EGF) in the regulation of signaling pathway mediating IL-1α (IL-1 alpha) expression in cervical epithelial cells.** Expressed IL-1α can then bind to its cognate receptor, interleukin-1 receptor type I (IL-1RI) to recruit interleukin-1 receptor accessory protein (IL-1RAP) on same cell leading to the activation of IL-1α signaling pathway (IL-1a pathway).

This study identifies PGE_2_ and EGF within SP as possible ligands responsible for SP-mediated induction and secretion of IL-1α in cervical cells. Furthermore, this study provides evidence for pathways that are used by SP to induce the production of pleotropic pro-inflammatory cytokine IL-1α in normal and neoplastic cervical epithelial cells following coitus. These pathways are summarized schematically in Figure 8 and show that SP activates EP2/EGFR/PI3 kinase-Akt signaling pathways to induce IL-1α expression in cervical cells. The secreted IL-1α can in turn regulate in an autocrine or paracrine manner through its cognate receptor the surrounding cells and promote inflammation and tumorigenesis.

## Materials and methods

### Ethics statement

Ethic approval for this study was obtained from the University of Cape Town Human Research Ethics Committee (HREC/REF: 067/2011). Written informed consent was obtained from all individuals before sample collection.

### Chemicals and reagents

Cell culture media, penicillin-streptomycin, and fetal-calf serum were purchased from Highveld Biological (PTY) Limited (Cape Town, South Africa). Bovine serum albumin (BSA), phosphate buffered saline (PBS), and Trizol® were all purchased from Sigma Chemical Company (Cape Town, South Africa). The chemical inhibitors: AG1478, epidermal growth factor receptor (EGFR) kinase inhibitor; LY294002, phosphoinositide-3-kinase (PI3K) inhibitor; SC560, prostaglandin synthase-1 (PTGS-1) inhibitor; NS398, prostaglandin synthase-2 (PTGS-2) inhibitor; and PD98059, extracellular signal-regulated kinases 1/2 kinase (ERK1/2) inhibitor were purchased from Calbiochem (Merck, Darmstadt, Germany). Prostaglandin E_2_, Butaprost, PGE_2_ receptor antagonist (AH6809), and human recombinant epidermal growth factor (EGF) were purchased from Sigma Chemical Company (Cape Town, South Africa). Quantikine® Human IL-1α ELISA kit was purchased from R&D Systems, Minneapolis, USA. Polyclonal goat anti-IL-1α (sc-1253) and biotin conjugated secondary donkey anti-goat IgG antibody (sc-2042) were purchased from Santa Cruz Biotechnology. Streptavidin-biotin peroxidase complex and 3.3’-diaminobenzidine were all purchased from Dako North America Incorporation, USA. Total PKB (Akt) (9272 s) and phosphorylated PKB (S473) antibodies were purchased from Cell Signaling Technology. Pierce® BCA Protein Assay Kit and SuperSignal® West Pico Chemiluminescent Substrates were purchased from ThermoScientific, Rockford, USA and PVDF membrane was purchased from GE Healthcare, Amersham, United Kingdom.

### Semen donors and preparation

Semen was collected from 10 healthy male volunteers attending the Andrology Laboratory of the Reproductive Medicine unit at Groote Schuur Hospital, Cape Town, South Africa. All donors had at least 72 hours of total sexual abstinence (self-reported) prior to ejaculation. Ejaculates were collected in sterile specimen jars following a voluntary self-masturbation. Parameters such as sample volume, sperm number, sperm concentration, peroxidase-positive leukocytes, pH and viscosity were noted and compared with the 2010 WHO (World Health Organization) reference values for human semen characteristics [76]. Samples with below average parameters were excluded from the study. All samples were processed within 30 minutes of collection. The individual ejaculates were transported to the laboratory and were pooled. Seminal plasma (SP) was isolated from the pooled ejaculate by centrifugation at 15000 g for 20 minutes. Seminal plasma was then aliquoted (200 μL) and stored at −80°C until required. Prior to use seminal plasma was thawed on ice and diluted in sterile filtered serum free medium to use at a concentration of 1:50. SP has been shown to exert no toxic effect on HeLa cell viability up to and including concentration used in this study [4,77].

### Cervical tissue collection and processing

Cervical cancer tissue specimens were obtained at the time of surgery or biopsy from patients (Ca.1-Ca.18) who were attending the Gynecologic Oncology Clinic at Groote Schuur Hospital (Cape Town) and who had previously been diagnosed with pre or invasive carcinoma of the cervix. Diagnostic cytology report was defined according to the Bethesda System for reporting cervical cytology [78] while pathological staging was defined according to the revised International Federation of Gynecology and Obstetrics (FIGO) staging for carcinoma of the cervix [79] upon physical examination. Patient age ranged between 29–62 years, with a median age of 41 years. The extent of invasiveness of carcinoma biopsies are presented in Table 1. Punch biopsies were taken from the lesion by an experienced gynecologist with a special interest in gynecology oncology. Samples were immediately transported in PBS on ice to the laboratory where they were divided into experiment and control and then serum starved overnight prior to stimulation with seminal plasma. Histologically normal cervical tissues were collected from women (N1-N12) undergoing Wertheim’s hysterectomy for benign gynecological malignancies at Groote Schuur Hospital (Cape Town). Patient age ranged between 37–73 years with a median age of 50.5 years. Cervical (approximately 3 × 4 mm) tissue samples collected at the time of surgery were immediately transported in PBS on ice to the laboratory where they were chopped into smaller pieces and divided into experiment and control before being serum starved overnight prior to SP stimulation.

**Table 1:** Histological typing, extent of invasiveness, and FIGO staging of carcinoma biopsies

**Sample identification**	**Histological type**	**FIGO staging**
**Ca.2-Ca.10, Ca.12-Ca.15**	Squamous carcinoma	0; carcinoma in-situ
**Ca.5,Ca.16**	Squamous carcinoma	IA; moderately differentiated
**Ca.1,Ca.17**	Squamous carcinoma	IA_1_; moderately differentiated
**Ca.11**	Adenocarcinoma	IB1; well differentiated
**Ca.18**	Squamous carcinoma	IB1; moderately differentiated
**Ca.4**	Squamous carcinoma	IIIB; moderately differentiated

### Cell culture and treatments

HeLa-S3 (HeLa) authenticated and verified as cervical adenocarcinoma cells positive for HPV-18 sequence with normal levels of pRB (retinoblastoma) and low levels of p53 tumour suppressor, were purchased from BioWhittaker (Berkshire, UK). Cells were routinely maintained in DMEM nutrient mixture F-12 with Glutamax-1 and pyroxidine supplemented with 10% fetal bovine serum (FBS) and 1% penicillin-streptomycin (500 IU/ml penicillin and 500 μg/ml streptomycin) at 37°C and 5% CO_2_ (v/v). For experiments, HeLa-S3 cells were seeded in medium supplemented with 10% FBS at density of 2 × 10^5^ cells in 3 cm diameter tissue culture dishes and allowed to attach and grow overnight after which cells were serum starved by incubating in serum free medium for 24 hours. Cells were then treated with vehicle (PBS) or SP at a dilution of 1:50 or butaprost [5 μM] or PGE_2_ [300 nM] or EGF [10 ng/ML] for 4, 8, 16, and 24 hours. For receptor blockade and inhibitor experiments, serum starved cells were treated with receptor antagonist/inhibitors alone or SP (1:50 dilution) alone or together for 4 or 16 hours. The antagonist and inhibitors used and their final concentrations were: EP2 receptor antagonist [AH-6809; 20 μM], inhibitors of EGFR [AG-1478; 100 nM], PI3 kinase [LY-294002; 25 μM], PTGS1 [SC-560; 15 μM], and PTGS2 [NS-398 8 μM]. The concentrations of chemical inhibitors used in this study were determined empirically by titration as described [80-83]. At the concentration and time used, the antagonist or inhibitors showed no adverse effects on cell viability when stained with 0.4% trypan blue dye. Fold increases was calculated by dividing the values obtained from the SP/SP-inhibitor treatments by the vehicle/vehicle-inhibitor treatments.

### Real –Time quantitative RT-PCR

Real-time quantitative RT-PCR was performed to determine the expression of IL-1α in cervical carcinoma biopsies and normal cervical tissue and also to assess the effects of ligands or chemical inhibitors on SP mediated induction of IL-1α in HeLa S3. RNA was extracted from neoplastic cervical tissue (adenocarcinoma Ca.11 and squamous cell carcinoma Ca.1 - Ca.10 and Ca.12 - Ca. 18), normal cervix (N1-N12), and HeLa S3 cells using Trizol-reagent as per the manufacturers’ instruction and reversed transcribed as described previously [10]. Real-time quantitative RT-PCR reaction was carried out on an Illumina ECO™ quantitative RT-PCR machine and detected using SYBR green (Bioline, Celtic Molecular, Cape Town, South Africa) incorporation during PCR reaction. Each sample was plated in duplicate and a no template control (H_2_0) was also included. A melt curve was performed for each PCR reaction and purity of PCR product determined by a single peak. Glyceraldehyde 3-phosphate dehydrogenase (GAPDH) and Human cyclophilin A (HCYPA) were included as reference genes in each experiment. These genes (GAPDH and HCYPA) were the two most stable genes of all five reference genes (Eukaryotic translation elongation factor 1 alpha 1 (EEF1A1), actin, β-actin, GAPDH and HCYPA) analyzed for HeLa S3 and cervical tissue when their average expression stability value M was calculated using geNorm in qbase + 2.5.1 software (Biogazelle, Belgium). All relative expressions were calculated using the comparative C_t_ relative to an endogenous control of HeLa cell cDNA included in each experiment. The sequences of IL-1α, GAPDH, and HCYPA primers used are presented in Table 2. Data are presented as fold increase as determined by dividing the relative expression of SP treated group by that of the control. Data are presented as mean ± SEM.

**Table 2:** List of real-time quantitative RT-PCR primers

**Gene**	**Primers**
**IL-1α**	5′-TGTATGTGACTGCCCAAGATGAA
	3′-CTACCTGTGATGGTTTTGGGTATC
**GAPDH**	5′-ACAGTCAGCCGCATCTTC
	3′- GTCCTTCCACGATACCA
**HCYP-A**	5′-CCCACCGTGTTCTTCGACAT
	3′-CCAGTGCTCAGAGCACGAAA

### Enzyme linked immunosorbent assay

Quantikine® Human IL-1α ELISA kit was used to assess IL-1α protein expression. The experiment was done on HeLa S3 cells seeded at a density of 5 × 10^5^ in 3 cm tissue culture dishes and serum starved overnight. The cells were then treated with SP (1:50) or PBS (control) for 16 and 24 hrs. Cells were lysed as described previously [10] and total protein quantified using Pierce® BCA Protein Assay Kit. Expressed cellular IL-1α protein was determined from the total protein in the lysate. Data are presented as fold change over control treated, which was calculated by dividing the amount of IL-1α measured in SP treated cells at the different time points by the amount measured in their respective controls. Data are presented as mean ± SEM of six independent experiments.

### SDS-PAGE and Western blot analysis

Immunoblot analysis was performed on solubilized cell lysates of cultured HeLa cells that were initially separated on a 10% SDS-PAGE gel that was run at 100 volts. Thereafter, separated proteins in the gel were transferred onto PVDF membrane and subjected to immunoblot analysis. Membranes were incubated in 10 mL of blocking buffer (PBS, 0.1% Tween-20 with 5% w/v nonfat dry milk) on a shaker for 1 hour at room temperature (RT), after which membranes were washed three times for 10 minutes each with 15 mL PBS, 0.1% Tween-20 followed by an overnight incubation with rabbit anti-PKB (AKT) and anti-P-PKB (AKT) (1:1000 dilution) at 4°C with gentle shaking. Thereafter, membranes were washed three times for 10 minutes each with 15 mL of PBS, 0.1% Tween-20, incubated for 1 hour with anti-Rabbit HRP-conjugated secondary antibody (1:5000 dilution) at RT with gentle shaking and washed again three times with 15 mL PBS, 0.1% Tween-20. Protein detection was done by chemiluminescence; membranes were incubated with SuperSignal® West Pico Chemiluminescent Substrate (1 mL of SuperSignal West Pico Luminol/Enhancer Solution and 1 mL SuperSignal West Pico Stable Peroxide Solution) for 5 minutes at RT and proteins viewed with BioSpectrum™ 500 Imaging System (Ultra-Violet Products [UVP] Limited, Cambridge, UK). Densitometry on visualized protein bands was done using ImageJ version IJ 1.46r (http://www.imagej.nih.gov/ij/). Akt phosphorylation was calculated by dividing the value obtained from phosphorylated Akt channel by the value obtained from total Akt channel and expressed as fold above vehicle controls. Data are presented as mean ± SEM of three independent experiments.

### Immunohistochemistry

Immunohistochemistry was done on archival cervical blocks (Normal n = 5, squamous cell carcinoma n = 4 and adenocarcinoma n = 4) obtained from the Department of Anatomical Pathology, University of Cape Town. Briefly, sections were deparaffinized and rehydrated by immersing in xylene twice for 5 minutes, 100% ethanol twice for 5 minute, 95% ethanol for 5 minutes, 70% ethanol for 5 minutes, 50% ethanol for 5 minutes and rinsed with water. Antigen retrieval was done by pressure cooking for 2 minutes in 0.01 M sodium citrate pH 6. Sections were blocked for endogenous peroxidase by incubating with 3% Hydrogen peroxide in methanol on a rocker at RT for 30 minutes and then rinsed with water followed by 1× TBS (50 mM Tris–HCl, 150 mM NaCl at pH 7.4). Sections were blocked using 5% normal donkey/goat serum diluted in TBS after which tissue sections were incubated with polyclonal goat anti-IL-1α (1:200) antibodies at 4°C for 18 hours. After incubation, tissue sections were then washed in TBS twice for 5 minutes each followed by incubation with biotinylated donkey anti-goat secondary IgG antibody at dilution of 1:500 at RT for 30 minutes. Tissue sections were then further incubated with streptavidin-biotin peroxidase complex (1:50) at RT for 30 minutes. Controls were incubated with biotinylated IgG secondary antibody only. Color reaction was developed by incubating with 3.3’-diaminobenzidine. Tissue sections were counterstained in aqueous hematoxylin, before mounting and coverslipping. Images were visualized and photographed using a Carl Zeiss laser scanning microscope LSM 510 (Jena, Germany).

### Statistical analysis

All data in this study were analyzed by t-test or one-way ANOVA using Graph Pad Prism 5.0 software (GraphPad Software Inc., San Diego, CA). Paired T-tests were conducted on the untransformed means of the replicates between SP and control. Unpaired T-tests were performed on SP versus SP and inhibitor after conversion to fold increases. One-way ANOVA was used as an additional tool to determine the significant difference between various time points for IL-1α by real-time PCR in response to SP.

## Competing interests

The authors declare that they have no competing interests.

## Authors’ contributions

AOA collected all tissue explants used in this study, performed real-time RT-PCR, western blot, ELISA, immunohistochemistry analysis and helped to draft the manuscript. In addition, AOA and KJS performed data acquisition, analysis and interpretation. KJS and AAK conceived the study and participated in its design and coordination and helped to draft the manuscript. All authors have read and approved the final manuscript.
